# Ebstein anomaly associated with retinal venular dilatation, migraine, and visual snow syndrome: a case report

**DOI:** 10.1186/s12886-022-02288-z

**Published:** 2022-02-14

**Authors:** P. T. V. M. de Jong, E. F. Thee, B. Straver

**Affiliations:** 1 Department of Ophthalmology, Amsterdam University Medical Center, AMC, Meibergdreef 9, 1105 AZ Amsterdam, The Netherlands; 2 Department of Retinal Signal Processing, The Netherlands Institute of Neuroscience, KNAW, Amsterdam, The Netherlands; 3 Department of Ophthalmology, Erasmus University Medical Center, Molewaterplein 40, 3015 GD Rotterdam, The Netherlands; 4 Department of Epidemiology, Erasmus University Medical Center, Rotterdam, The Netherlands; 5 Department of Pediatric and Congenital Cardiology, Amsterdam University Medical Center, AMC, Meibergdreef 9, 1105 AZ Amsterdam, The Netherlands

**Keywords:** Case report, Ebstein anomaly, Migraine with aura, Retinal venular dilatation, Visual snow syndrome

## Abstract

**Background:**

To present a case with Ebstein anomaly, a rare congenital heart disorder, with ophthalmological and neurophthalmological signs and symptoms. To date, retinal venous dilatation and visual snow syndrome have not been previously been published in this anomaly.

**Case presentation:**

A 10-year-old white girl was diagnosed with Ebstein anomaly. From age 12, she regularly suffered from migraines with auras and photophobia. At age 16 she started to see short-term bouts of white snow, that after a year were present all day. At age 20, she was found to have a decreased retinal arteriovenous ratio.

**Conclusions:**

Retinal arterial tortuosity and venular dilatation are common in congenital heart disease but have not been described in Ebstein anomaly, nor has the visual snow syndrome.

## Background

Ebstein anomaly (EA) [1] is a congenital myopathy of the right cardiac ventricle due to an abnormal tricuspid valve. There are many variations in its deviant tricuspid anatomy that usually causes tricuspid blood regurgitation. As a result, there is a high incidence of right ventricular dysfunction and arrhythmias [2, 3]. About 1% of live-born children has a congenital heart disease (CHD) and EA accounts for 1% of all CHDs, leading to a prevalence of 0.05 per 10.000 live births [4]. It usually occurs sporadically and rarely is familial [4]. So far, we found one publication on ocular abnormalities in EA [5]. We will describe three more symptoms and signs and show why we hypothesize that they are associated with this anomaly.

## Case presentation

A 20-year-old female received the diagnosis EA at the age of 10 years after becoming short of breath while playing hockey. At rest, her oxygen saturation was 97% but with exercise it dropped to 78%. From age 12, she had migraine attacks, usually just before or during her period, accompanied by auras, slight headache and mild photophobia. One day in school at age 16, white, partly transparent, sometimes shimmering dots appeared in front of both eyes. At times, these dots looked like “a trembling image over distant warm asphalt.” Initially, the spots appeared several hours a day, but soon they were present from rising in the morning to bedtime, and were more noticeable in bright light, during sleep deprivation or alcohol ingestion but not with physical exertion. The spots were most pronounced in the peripheral field of vision and hardly visible in the center. She had some photopsia’s but no palinopsia (after-images). The patient has never squinted or received occlusion therapy. She never had ocular or other surgery and did not complain of diplopia or night blindness. She never needed glasses to see properly. She visited an ophthalmologist and a neurologist who found no abnormalities and had no explanation for her complaints. Her brain MRI was normal. Seven months before her arrival at our clinic, she noticed slight tremors, hypoesthesia, and loss of strength in her right arm for a week. She was right-handed. Her medical history otherwise was normal and in particular did not mention depression, epilepsy, loss of consciousness, tinnitus, dizziness or arrhythmia symptoms.

Her two younger brothers were healthy and there were no eye disorders in the family other than cataracts and mild refractive errors. Her mother had not noticed any abnormalities during pregnancy or birth. Other than oral contraceptives, she did not use any medications or drugs; she smoked 10 cigarettes and consumed four alcoholic drinks a day.

### Cardiac examinations

A cardiac MRI at age 10 and 18 years, showed no abnormalities like an abnormal cardiac vascular pedicle, aortic coarctation or a Chiari network in her right ventricle. Echocardiography in 2021, revealed an apically displaced tricuspid valve, typical of EA. Minor regurgitation was present at a low estimated ventricular pressure, given the absence of pulmonary hypertension. Systemic venous return was normal, as were respiratory collapse and central venous pressure and also antegrade pulmonary blood flow was normal. A small atrial septal defect led to left-right shunting at rest, that became bidirectional with exertional echocardiography, in which the oxygen saturation decreased from 97 to 80%, without increase in tricuspid regurgitant flow. Her blood pressure, kidney functions, hemoglobin, hematocrit, mean corpuscular volume, and platelet counts were normal.

### Eye examinations

**Fig. 1:**
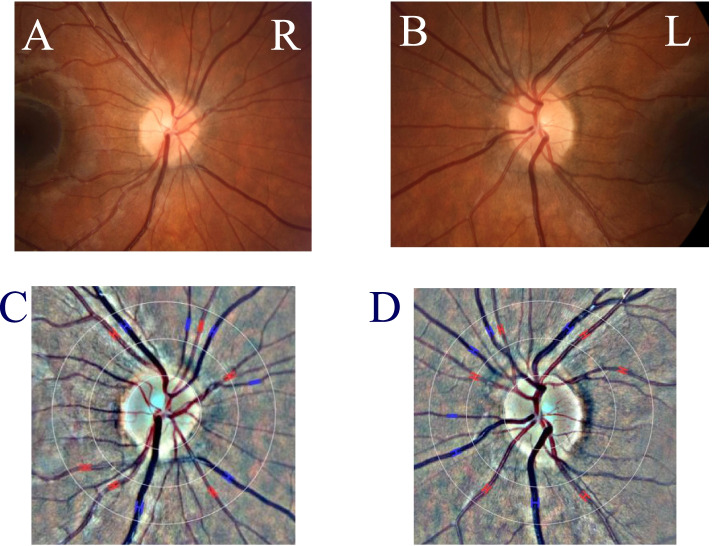
**A** Colour image of the right optic nerve head from which major retinal vessels arise. The arterioles are light red in colour, the venules dark red. **B** Similar image of left optic nerve head. **C** Digitized image of A with concentric circles drawn around optic nerve to calculate the arteriovenous ratio. This ratio was 0.648. **D** Similar image of B. This ratio was 0.670

Visual acuity was 1.25 in her right and 1.0 in the left eye, both with Sph + 0.25. There were normal anterior eye segments and clear lenses in both eyes, normal direct and consensual pupillary reactions with no relative afferent defect and normal optic discs with a vertical cup/ disk ratio 0.4 in both eyes. After mydriatic drops, the macula and retinal periphery were normal in each eye. Wide venules (Fig. 1) were visible as the only deviation on ophthalmoscopy. Below the right optic disc, a venule branched off at a right angle and there was no marked vascular tortuosity. Confrontational visual field testing was normal, as was the AO-HRR colour vision test, taken separately for each eye. Stereo vision with the TNO test was perfect with 60 arcsec. Optical coherence tomography was normal in both eyes. We calculated that the arterio-venous ratio (AVR) of the retinal vessel diameters was 0.684 in the right and 0.670 in he left eye (Fig. 1). In addition to the already known migraine, we diagnosed dilatation of the retinal venules and visual snow syndrome.

## Discussion and conclusions

The dilatation of the retinal venules and the neurophthalmological visual snow syndrome in this patient have not been described in EA before. This is all the more remarkable because CHD has for over 50 years been known to be associated with conjunctival hyperemia, tortuosity of the retinal arterioles and distension of its venules [6] as well as a cyanotic fundus hue and papilledema [7].

Until computerized systems were developed, calculation of AVR was problematic [8]. AVR calculation usually takes place in premature infants or in older populations with cardiovascular problems. In full-term newborns, the AVR is 0.66 [9]. Normotensive persons accompanying patients to a hospital, had an AVR at age 10–20 of 0.870, and the AVR decreased with every decade by about 0.02 to 0.761 at age 69 years [10]. The AVR of our patient was thus clearly low for her age and given the absence of systemic hypertension, we attribute this to her EA. Nearly all patients with EA have a patent oval foramen, [11] leading to right–left (RL) shunting of blood. As a result, hypoxia and cyanosis may occur leading to erythrocytosis. Venules can dilate, arterioles less so. The exact mechanism of retinal vessel remodeling is still uncertain [12]. Patients with cyanotic CHD have a lower retinal vascular density in the peripapillary capillary plexus on optical coherence tomographic angiography than those with similar non-cyanotic disease. This vascular density for both patient groups was lower than in healthy control persons and was positively correlated with oxygen saturation and negatively with hematocrit [13].

The life-time prevalence of migraine in the general population is 13% for men and 33% for women [14]. Migraine-like visual auras are attributed to neuronal hyper excitability that in its turn triggers cortical spreading depression [15]. In patients with CHD without RL shunting, the prevalence of migraine went up to 43%, but in EA to 67% [11]. Authors hypothesized that bioactive substances are normally removed by the lungs during the pulmonary circulation. Due to the RL shunt, some of these substances immediately would enter the cerebral circulation. Severe migraines may indicate operative closure of an open oval foramen [11].

Until about 2013, one considered visual snow to be a migraine variant [16] or a “hallucinogen persisting perception disorder,” a side effect of recreational drug use [17, 18]. The first description of visual snow among cocaine users dates back to 1978 [19]. These hallucinations were called snow lights, analogous to sunlight reflecting from snow crystals. In a letter with retrospective data from 20 patients, authors stated that visual snow should be considered a new visual syndrome. There was a 2:1 female sex predominance, one third had persisting palinopsia, one quarter migraine with aura and 15% complained about tinnitus [18]. In later articles, mainly from the neurological angle, entoptic phenomena, photopsia, photophobia and nyctalopia were added as well as anxiety and depression as comorbidities [20]. Currently, visual snow syndrome is poorly understood. Twenty persons showed hyper metabolism on [18F]-2-fluoro-2-deoxy-D glucose positron emission tomography as well as an increase in the extra striate visual cortex volume [21]. Recently, a MRI study demonstrated in 24 patients increased gray cerebral matter volume in the primary and secondary visual cortex as in a cerebellar crus [22]. It is hypothesized that visual snow syndrome is, like migraine, due to hyper excitability of the visual cortex and is a processing disorder of the higher optical functions [23]. There is no proven cure for this syndrome. When both migraine and visual snow are associated with EA, and are due to hyper excitability of the cerebral cortex, one would expect that there should be changes in the electroencephalogram (EEG). The more, because 8 of 12 patients with CHD had an abnormal EEG, that did not improve after cardiac surgery [7]. However, all 20 patients in a recent study on visual snow syndrome had normal EEGs and normal visually evoked cortical potentials [21].

The neurologist who saw the patient did not comment on whether he thought of a cerebrovascular accident because of the hypoesthesia and loss of strength in her right arm. However, we have to keep such an accident in mind because 9% of 968 patients with EA had a stroke [24]. That is why we urgently advised the patient to stop smoking. She was reassured that she was not imagining the visual snow, but regretted that nothing could be done about it yet.

We think we can conclude that CHD and the EA might also be considered as a cause of visual snow or dilated retinal venules.

## Data Availability

The datasets used and/or analyzed during the current study are available from the corresponding author on reasonable request.

## Abbreviations

AO: American Optical
HRR: Hardy Rand Rittler
AVR: Arteriovenous ratio
CHD: Congenital heart disease
EA: Ebstein anomaly
EEG: Electroencephalogram
MRI: Magnetic resonance imaging
RL: Right-left
TNO: Toegepast Natuurwetenschappelijk Onderzoek
